# Enhancing Fire Resistance and Mechanical Properties of Wood Strand Boards by Impregnation with Sodium Bicarbonate and Sodium Borate

**DOI:** 10.3390/polym17212943

**Published:** 2025-11-04

**Authors:** Roger Pedieu, Aziz Bentis, Bernard Riedl, Xiang-Ming Wang, James Deng, Flavia Lega Braghiroli, Ahmed Koubaa

**Affiliations:** 1 Centre de Recherche sur les Matériaux Renouvelables, Université Laval, Pavillon G-H. Kruger 2425, rue de la Terrasse, Québec, QC G1V 0A6, Canada; pedieu_1999@yahoo.com (R.P.); bernard.riedl@sbf.ulaval.ca (B.R.); 2 Institut de Recherche sur les Forêts (IRF), University of Québec in Abitibi-Témiscamingue (UQAT), 445 Boul. de l’Université, Rouyn-Noranda, QC J9X 5E4, Canada; aziz.bentis@uqat.ca (A.B.); flavia.braghiroli2@uqat.ca (F.L.B.); 3 FPInnovations, Division Forintek, 319 rue Franquet, Québec, QC G1P4R4, Canada; xwang0529@hotmail.com (X.-M.W.); james.deng@fpinnovations.ca (J.D.)

**Keywords:** oriented strand boards, flame retardancy, mechanical properties, sodium bicarbonate, sodium borate, sustainability, halogen-free flame retardants

## Abstract

The development of halogen-free flame-retardant formulations for wood-based panels is a promising strategy to improve both fire safety and environmental performance. In this study, oriented strand boards (OSB) were impregnated with aqueous solutions of sodium borate (SBo) and sodium bicarbonate (SBi) to evaluate their combined effects on fire resistance and mechanical properties. Fire performance was assessed using the ASTM D3806 small-scale tunnel test, while mechanical and physical properties were measured according to ASTM D1037. Significant improvements in fire performance were observed: mass loss (ML) during flammability testing decreased by 38% (from 6.9% to 4.3%), flame spread speed (FSS) was reduced by more than 50% (from 6.8 to 3.3 mm/s), and after-flame times (AFT) dropped from 17.2 s to 0 s. Thermogravimetric analysis (TGA) further confirmed enhanced thermal stability, with increased char residue (from 16.9% in untreated boards to 31.5% in treated ones). Mechanical testing revealed a 16% increase in internal bond (IB) strength (from 0.44 to 0.51 MPa), while modulus of rupture (MOR) and modulus of elasticity (MOE) were only slightly affected (decreased by up to 4.2% and 3.6%, respectively). Interestingly, the two additives exerted contrasting effects: SBo reduced strength and bonding performance, whereas SBi improved internal bond strength and dimensional stability. The optimal balance was obtained with treatment P250-50 (250 g SBi and 50 g SBo), which combined enhanced fire resistance with acceptable mechanical integrity. Overall, the results demonstrate that the synergistic use of SBo and SBi offers an effective halogen-free approach to simultaneously enhance the fire resistance and mechanical performance of OSB panels, highlighting its potential for industrial applications.

## 1. Introduction

Wood is a naturally renewable and durable material that is extensively used in construction because of its aesthetic appeal, lightweight nature, flexibility, ease of processing, and excellent mechanical properties. Its composition is primarily organic, consisting of cellulose, hemicellulose, and lignin, which contribute to its remarkable durability and versatility in building applications [1,2,3]. However, a significant limitation of wood is its inherent flammability, which poses a considerable challenge for its use in industrial and institutional applications, particularly in buildings where fire safety is a critical concern [4,5,6].

The sources of fires in buildings often include electrical malfunctions, open flames, and combustible materials. In wood-based construction, these sources can lead to rapid fire propagation owing to the high flammability of cellulose, the main organic component of wood. The decomposition of cellulose produces volatile gases that contribute to flame spread, posing severe risks in residential and commercial settings [7,8]. Thus, enhancing the fire resistance of wood without compromising its intrinsic physical and mechanical properties is a critical research objective [9,10,11]. Fire-retardant treatments have been extensively explored. Traditional methods often involve the use of halogenated compounds, which, despite their effectiveness, pose environmental and health concerns due to the release of toxic gases during combustion [12,13]. Recent studies have focused on halogen-free treatments, which are safer alternatives for industrial applications. Magnesium compounds, such as MgSO_4_·7H_2_O, which are also employed in the treatment of wood to render it fire-resistant, undergo endothermic decomposition, releasing water vapor or CO_2_ in the process. This process absorbs a proportion of the heat of combustion, concomitantly reducing the temperature of the wood. The inert gases released during this process dilute the flammable gases present in the condensed phase [14]. Other promising systems include boron, phosphorus, and nitrogen compounds, which act through char promotion and flame inhibition [15,16,17,18]. Boron compounds, including sodium borate and boric acid, have been identified as effective fire retardants owing to their ability to promote char formation and inhibit flame spread [19,20,21]. These compounds function primarily through thermal decomposition, forming a protective layer that inhibits combustion and reduces heat transfer during a fire event [22]. Borate compounds, such as disodium octaborate tetrahydrate (DOT), are widely recognized for their ability to diffuse into wood and interact with cell wall polymers, providing biological protection and fire resistance without significantly compromising mechanical properties. In some cases, slight improvements in modulus of rupture (MOR) and modulus of elasticity (MOE) have even been reported when treatment levels are compatible with adhesive systems, including phenol–formaldehyde and melamine–urea–formaldehyde resins [23,24]. Borates also act synergistically with other flame-retardant systems by promoting char formation, delaying pyrolysis, and suppressing volatile release, while maintaining structural integrity in composite boards [25]. Boron-based flame retardants are recognized as one of the most efficient candidates for producing fire-retardant wood and particleboards [26]. A significant study demonstrated that incorporating colemanite, a naturally occurring boron mineral, into particleboards results in improved fire resistance and thermal degradation properties. This research suggests that colemanite could serve as a cost-effective alternative to traditional boron-based FRs, providing similar or enhanced performance while reducing the environmental impact [27]. The thermal degradation of wood treated with boron compounds has been shown to produce non-flammable gases, such as ammonia and water vapor, which dilute combustible gases and lower the surface temperature of the material, further enhancing fire resistance [28]. Additionally, the use of boron-based FRs has been linked to improved mechanical properties of wood composites. For instance, the addition of boron-containing oligomers to wood coatings has been shown to enhance both flame retardancy and mechanical strength, making them suitable for application in construction and furniture [29]. The synergistic effects of combining boron compounds with other flame retardants, such as phosphorus-based systems, have also been explored, revealing that these combinations can lead to superior flame-retardant properties compared with single-component systems [30,31]. Moreover, the application of boron-based FRs in laminated bamboo lumber has shown promising results, where the use of phenol-formaldehyde and melamine-urea formaldehyde adhesives in conjunction with boron compounds significantly improves the fire performance of the material [28].

Similarly, sodium bicarbonate has gained attention as a low-toxicity, halogen-free additive that decomposes to release CO_2_ and Na_2_CO_3_ during combustion [32,33]. This reaction not only dilutes oxygen but also contributes to the formation of a compact char layer, improving thermal stability with minimal impact on bonding or dimensional stability. Several studies have investigated the role of sodium bicarbonate (NaHCO_3_) as a fire-retardant additive in lignocellulosic materials. For example, Bakirtzis et al. [32] tested NaHCO_3_ at different loadings (5–20 wt%) on three Mediterranean forest species (Pinus brutia, Laurus nobilis, and Nerium oleander) using thermogravimetric analysis (TGA) and controlled furnace combustion experiments. The results showed that NaHCO_3_ shifted the onset of thermal degradation to lower temperatures, promoting earlier pyrolysis, while simultaneously delaying self-ignition. In fact, the time to ignition increased with NaHCO_3_ addition, reaching up to two-fold longer values compared to untreated samples, due to the release of H_2_O and CO_2_ during its decomposition. Combustion duration was also extended, particularly in *P. brutia*, and the char residue significantly increased, with values up to 20–25% higher than the untreated controls. Overall, these findings highlight the dual effect of NaHCO_3_, acting both as a promoter of early pyrolysis and as an inhibitor of flaming ignition and combustion, confirming its potential as an effective, halogen-free fire-retardant additive for lignocellulosic systems. Another promising approach involves the use of ammonium polyphosphate (APP) and tris(2,3-dibromopropyl) isocyanurate (TBC) as synergistic flame retardants in strandboard. Tian et al. [34] reported that the addition of APP enhanced thermal stability and promoted early char formation, while TBC exerted minimal influence on resin curing and preserved mechanical strength. Notably, their optimal hybrid formulation achieved a MOR of 30.5 MPa, an MOE of 4159 MPa, and an internal bond (IB) of 0.93 MPa, values that are significantly higher than those of boards treated with APP alone. Combustion testing further confirmed the synergistic action, as the combined APP–TBC system produced a denser and more cohesive char layer. These findings underscore that carefully designed hybrid systems can simultaneously enhance both flame retardancy and mechanical performance—an outcome also sought in the present work using SBi and SBo.

In our previous study, we demonstrated that impregnation could be an effective method for flame-retardant treatment of wood using boric acid [4]. The results showed that particleboards treated with 16% boric acid exhibited excellent fire-retardant performance, including reduced weight loss, slower flame spread, and shorter after-flame times. Additionally, the incorporation of white birch inner bark particles further enhanced fire resistance by decreasing weight loss during fire tests. Our findings also indicated that boric acid treatment improved the internal bond strength and reduced thickness swelling. At the same time, birch particles made a positive contribution to the overall performance without compromising the mechanical or physical properties. These results underscore the potential of boric acid as a highly effective and practical fire-retardant agent for industrial particleboard production. To the best of our knowledge, no previous study has systematically evaluated the effects of SBi and SBo on the performance of oriented strand boards (OSB). In particular, their synergistic use through an industrially compatible impregnation process has not been investigated for the simultaneous optimization of fire resistance, mechanical integrity, and dimensional stability. This work focuses on the impregnation of wood strands using a simple aqueous pressure process with non-toxic, low-cost salts that require no modification of existing OSB production lines. Impregnation with SBi and SBo modifies the chemical composition of wood strands throughout their cross-section, not merely at the surface. During hot-pressing, these salts migrate toward strand interfaces, where they influence resin curing, interfacial adhesion, and char formation during combustion. The aim is to enhance the fire resistance of OSB while maintaining its intrinsic mechanical and physical properties. Specifically, the objective is to determine how the interaction between SBi and SBo influences the fire-retardant, physical, and mechanical performance of OSB, and to identify optimal formulations that balance fire safety with structural integrity. The simplicity and industrial scalability of this halogen-free treatment process make it particularly attractive for large-scale applications, while also minimizing the environmental footprint and aligning with current sustainability goals in the wood treatment industry.

## 2. Materials and Methods

### 2.1. Preparation of Treating Solution

Strands were obtained from a Louisiana Pacific (LP) OSB mill located in Maniwaki, QC, Canada. Sodium bicarbonate (NaHCO_3_, ≥99.7%, crystalline powder) was purchased from Fisher Scientific (Ottawa, ON, Canada), and Disodium tetraborate dehydrates (Na_2_B_4_O_7_·10H_2_O, ≥98%, Tim-Bor^®^ Professional, Nisus Corporation, Rockford, TN, USA) was used as the boron source. The treatment solutions consisted of binary mixtures of SBo and SBi at different concentrations (Table 1). For each formulation, the corresponding mass of SBo and SBi was weighed and dissolved in 80 L of distilled water. Mixing was carried out for 1 h using an electric rotary mixer to ensure complete dissolution.

### 2.2. Impregnation Procedure

Strands were treated by pressure impregnation, as per ASTM D1413 [35]. A vacuum (30 min) was first applied to evacuate air from the strands, followed by 2.5 bar pressure for 30 min to facilitate solution penetration. After impregnation, the strands were extracted from the treatment solution and subjected to initial drying at room temperature for 4 days, followed by further drying in a laboratory dryer until 2% moisture content was achieved.

### 2.3. Manufacturing Parameters

Boards with dimensions 760 mm × 760 mm × 11 mm, with a target density of 625 kg/m^3^, were manufactured using a 1000 mm × 1000 mm Dieffenbacher hot press equipped with a PressMAN control system (Alberta Research Council, Calgary, AB, Canada). Liquid phenol-formaldehyde (PF) (PF, 52% solids, Borden Casco-Resin, Toronto, ON, Canada) was used. A PF content of 5%, based on the oven-dry weight of wood strands, was used for the core and surface layers. Wax at a 0.75% proportion based on the oven-dry weight of the wood strands was used in the core and surface layers. After blending, the mats were manually formed in a frame before hot-pressing. In the present work, the strands were not oriented; consequently, only the modulus of elasticity and modulus of rupture in the major axis were measured. Boards were pressed at a platen temperature of 200 °C. The press closing time was 26 s, curing time was 240 s, press pressure was 180 KPa, and opening time was 60 s, in three steps. Boards were conditioned at 21 °C and 65% RH for four weeks to obtain equilibrium moisture content before testing.

### 2.4. Characterization of Physical and Mechanical Properties

Samples for testing mechanical and physical properties were prepared according to ASTM D1037-99 [36] specifications (ASTM Book of Standards, 2005). The modulus of elasticity (MOE) and modulus of rupture (MOR) were obtained from the average of four 314 mm × 75 mm samples for each particleboard. The internal bond (IB) was obtained from the average of five 50 mm × 50 mm samples for each particleboard. The thickness swelling (TS) after soaking the samples in water for 24 h was obtained from the average of two 150 mm × 150 mm samples for each particleboard. Linear expansion (LE) was obtained from the average of two 300 mm × 75 mm samples for each particleboard. LE was measured following desorption from 65% to 50% RH and adsorption from 50% to 90% RH. RH conditions were obtained using a Cincinnati Sub-Zero CSZ climatic chamber (WM–906–MP2H–5–SC/WC).

### 2.5. Fire Performance and Thermal Stability

The fire-retardant properties were measured according to the ASTM D3806-77a [37] Standard Test Method of Small-Scale Evaluation of Fire-Retardant Paints (2-Foot Tunnel Method) (Annual book of ASTM Standards 2001). Thermogravimetric analysis (TGA) was conducted using a TA Instruments Q50 system. Measurements were carried out under a nitrogen atmosphere with a flow rate of 40 mL/min. Samples weighing 10 mg were heated from 50 °C to 600 °C at a constant heating rate of 10 °C/min. The resulting thermogravimetric curves were used to evaluate the thermal stability and degradation behavior of untreated and treated boards.

### 2.6. Statistical Data Analysis

The tests were conducted using a randomized complete block design (RCBD) to select the most suitable board type. Two factors are considered in this study. The first was the weight of SBi in 4 L of water: 150 g vs. 250 g. This quantitative factor was considered qualitative in this study to facilitate the use of the Statistical Analysis System (SAS) (version 9.4; SAS Institute Inc., Cary, NC, USA). The second was the weight of SBo in 4 L of water, which was considered to be quantitative: 25, 50, and 75 g. Blocking is used to prevent the nuisance factor of known and controllable sources of variability. Six board types are generated. Each type was replicated thrice for a total of 18 particleboards. Three control (untreated) boards were also manufactured for comparison, using Dunnett’s test, which was specifically designed to compare all groups against a single reference group.

## 3. Results and Discussions

### 3.1. Mechanical and Physical Properties

#### 3.1.1. Modulus of Elasticity (MOE)

**Figure 1:**
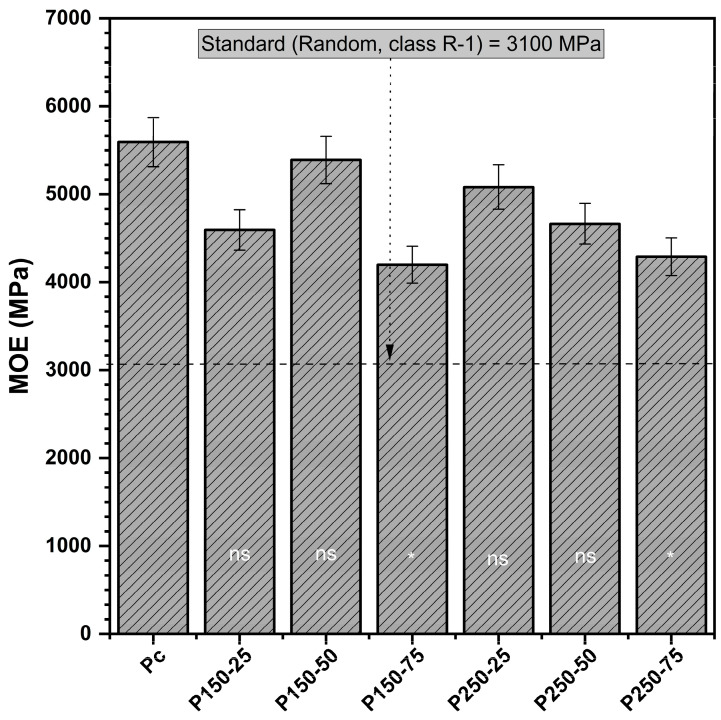
Modulus of elasticity (MOE) (mean values and standard deviation) [P_c_ = control board; P_x−y_ = description of treating solution: X g of sodium bicarbonate and Y g of sodium borate. The MOE of each board type is compared to controls using Dunnett’s test. Results are presented in the histogram as either ns (non-significant) or * (significant)].

All particleboards met the standard requirements for the modulus of elasticity (MOE) of R-1 type strand boards (Figure 1). The control boards averaged 5594 MPa, while treated boards ranged from 4199 to 5391 MPa, depending on formulation. Although ANOVA revealed no significant interaction between SBi and SBo, the effect of SBo alone was evident, with higher concentrations progressively reducing stiffness. For example, the MOE of the P250-75 formulation declined to 4261 MPa (−24% relative to control), while the P150-75 boards showed a similar reduction to 4199 MPa (−25%). In contrast, moderate formulations, such as P150-50 (5391 MPa) and P250-25 (5082 MPa), retained MOE values close to those of the control, indicating that stiffness losses are concentration-dependent rather than inherent to the treatment.

From a structural performance perspective, all values remain within the acceptable range for R-1 strand boards, suggesting that the negative influence of borate is tolerable at moderate loadings. These findings are consistent with earlier reports, which show that excessive borate incorporation can weaken load transfer within the wood matrix by disrupting hydrogen bonding between fibers. At the same time, lower concentrations preserve adequate stiffness [15,27].

#### 3.1.2. Modulus of Rupture (MOR)

**Figure 2:**
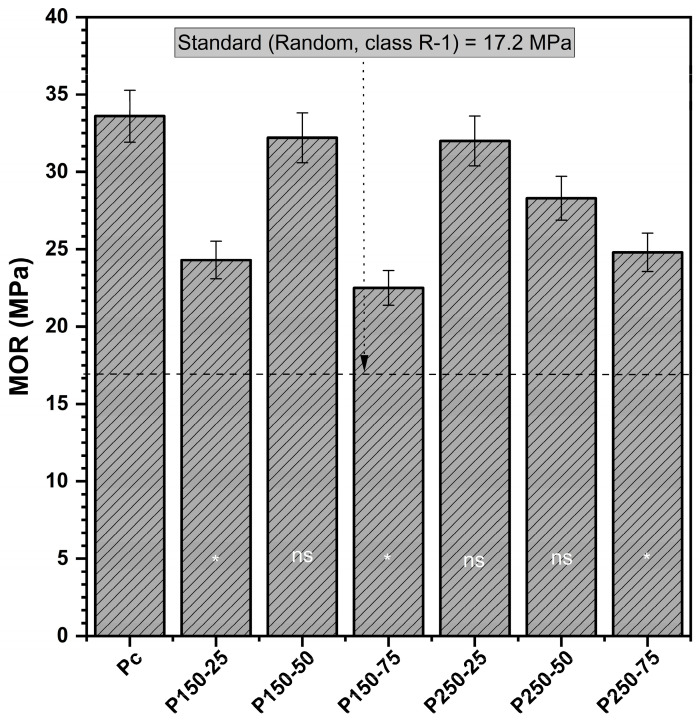
Modulus of rupture (MOR) (mean values and standard deviation) [P_c_ = control board; P_x−y_ = description of treating solution: X g of sodium bicarbonate and Y g of sodium borate. The MOR of each board type is compared to controls using Dunnett’s test. Results are presented in the histogram as either ns (non-significant) or * (significant)].

The modulus of rupture (MOR) of control boards averaged 33.6 MPa (Figure 2). A clear concentration-dependent decline was observed with increasing SBo loading. Boards treated with the highest borate content, such as P150-75 (22.5 MPa, −33%) and P250-75 (24.8 MPa, −26%), showed the greatest reductions relative to the control. In contrast, moderate formulations exhibited more favorable performance: P250-25 retained 32.0 MPa (−5%), and P150-50 reached 32.2 MPa (−4%), both values statistically comparable to those of the untreated board. These results indicate that excessive borate content compromises flexural strength, while balanced SBi–SBo ratios allow MOR values to remain within acceptable limits.

Dunnett’s test confirmed that only boards treated with the highest borate concentrations showed significant reductions in MOR (*p* < 0.05) compared to the control, whereas intermediate treatments did not differ statistically. The observed reductions can be explained by the interaction of borate salts with the wood cell wall and adhesive matrix, which may weaken fiber–matrix adhesion and reduce effective load transfer. Similar decreases in flexural strength at elevated boron loadings have been reported for particleboards and laminated composites [38,39,40], supporting the present findings. Nevertheless, all treated boards complied with the MOR requirements for R-1 strand boards, confirming that the treatment, even at high loadings, does not compromise structural standards.

#### 3.1.3. Internal Bond (IB)

**Figure 3:**
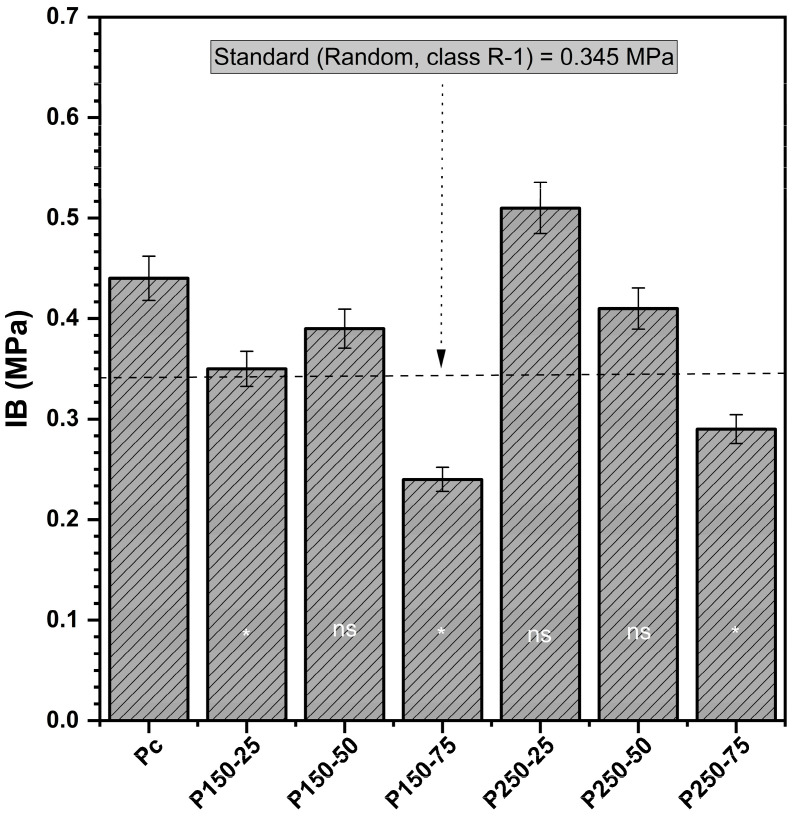
Internal bond (IB) (mean values and standard deviation) [P_c_ = control board; P_x−y_ = description of treating solution: X g of sodium bicarbonate and Y g of sodium borate. The IB of each board type is compared to controls using Dunnett’s test. Results are presented in the histogram as either ns (non-significant) or * (significant)].

Internal bond (IB) strength was highly sensitive to the combined effects of SBi and SBo (Figure 3). The control boards averaged 0.44 MPa, which meets the R-1 type strand board requirement. Boards treated with the highest borate concentration (P150-75) exhibited the lowest IB (0.24 MPa, −30% relative to control), confirming the antagonistic influence of borate on bond integrity. The ANOVA results further indicated a significant interaction between MBi and MBo (F = 11.04, *p* < 0.01), demonstrating that the effects of borate were moderated by bicarbonate content. Indeed, increasing the SBi content counteracted the weakening effect of SBo: the P250-25 formulation achieved a significantly higher IB (0.51 MPa, +16%) compared to the control.

Dunnett’s test confirmed these trends, showing that IB was significantly reduced at the highest SBo level (*p* < 0.05) but significantly improved in the P250-25 treatment. These results suggest that SBi promotes better inter-fiber bonding by buffering the resin curing process or reducing localized stress concentrations. At the same time, high SBo disrupts matrix adhesion through interference with hydrogen bonding or increased brittleness of the cured interface. Such trends are consistent with previous studies, which report that boron-based additives can weaken adhesive interactions at elevated concentrations, whereas optimized hybrid systems preserve or even enhance bond performance [41].

#### 3.1.4. Thickness Swelling (TS)

**Figure 4:**
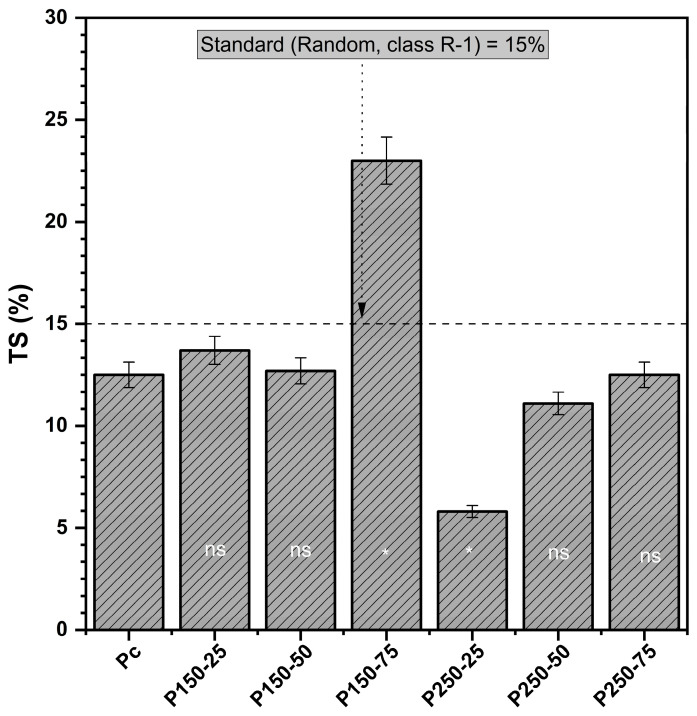
Thickness swelling (TS) (mean values and standard deviation) [P_c_ = control board; P_x−y_ = description of treating solution: X g of sodium bicarbonate and Y g of sodium borate. The TS of each board type is compared to controls using Dunnett’s test. Results are presented in the histogram as either ns (non-significant) or * (significant)].

Control boards exhibited a TS of 12.5% after 24 h water immersion (Figure 4), which is close to the upper limit of the R-1 strand board standard. The effect of chemical treatments on dimensional stability was strongly dependent on the relative concentrations of SBo and SBi. ANOVA confirmed a highly significant MBi × MBo interaction (F = 10.95, *p* < 0.01), indicating that the two salts act antagonistically when used in combination. At high borate levels, as in P150-75, swelling increased to 23% (+84% relative to control), reflecting the negative impact of SBo on water resistance at elevated concentrations. In contrast, Dunnett’s test showed that the P250-25 formulation achieved a significantly lower TS (5.8%, a 54% decrease compared to the control, *p* < 0.05), representing a substantial improvement in dimensional stability.

These results highlight a dual effect: while SBi at higher loadings (250 g) effectively reduced TS, possibly by forming carbonate deposits that limited water ingress, SBo at elevated concentrations exacerbated swelling, likely by interfering with adhesive penetration and creating microstructural pathways for water uptake. However, moderate combinations (e.g., P250-50, TS = 11.1%) yielded values comparable to those of the control, demonstrating that careful optimization can balance these opposing trends.

#### 3.1.5. Linear Expansion (LE)

**Figure 5:**
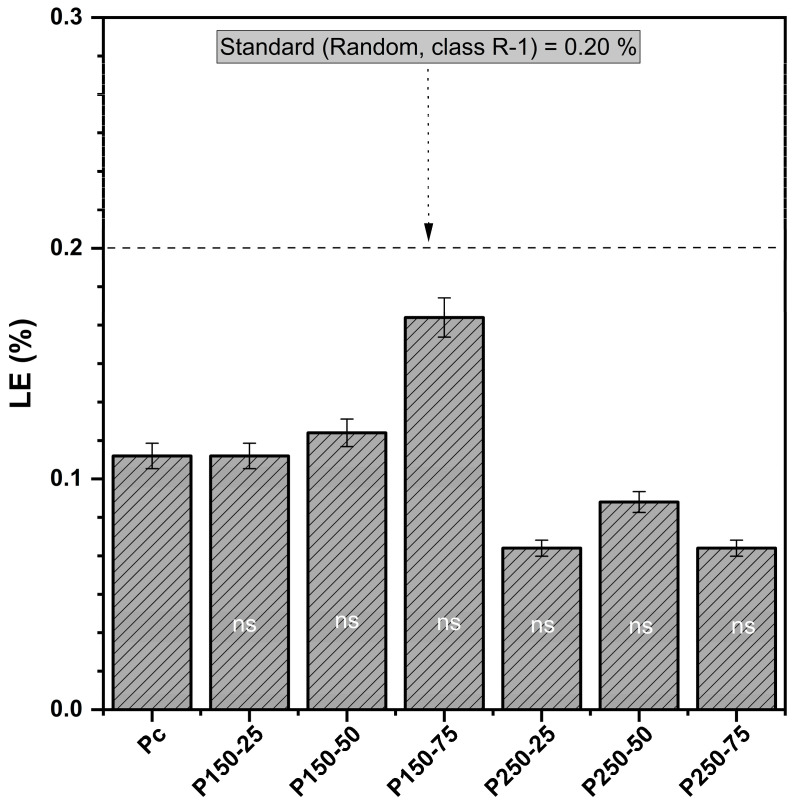
Linear expansion (LE) (mean values and standard deviation) [P_c_ = control board; P_x−y_ = description of treating solution: X g of sodium bicarbonate and Y g of sodium borate. The LE of each board type is compared to controls using Dunnett’s test. Results are presented in the histogram as either ns (non-significant)].

The control boards exhibited an average LE of 0.11%, while treated boards ranged from 0.07% to 0.17% (Figure 5). ANOVA results indicated no significant interaction between SBi and SBo (*p* > 0.05), suggesting that the simultaneous presence of both additives in the impregnation solution did not produce measurable synergistic or antagonistic effects on in-plane dimensional stability. Dunnett’s test similarly showed no statistically significant differences in LE between the treated and control board. Although statistical differences were not significant, a clear trend can be observed: formulations with higher SBi content generally exhibited slightly lower LE values (e.g., P250-25 and P250-75, both 0.07%), indicating a potential minor stabilizing influence of SBi. In contrast, the formulation containing the highest SBo concentration (P150-75) showed a marginally higher LE (0.17%, +55% compared to the control), although still within standard limits. These variations likely arise from subtle changes in hygroscopic behavior induced by the impregnation salts—SBi possibly promoting limited pore sealing via carbonate deposition. At the same time, while excess SBo may slightly increase hygroscopicity through residual borate compounds.

Overall, the results confirm that both SBi and SBo treatments preserve in-plane dimensional stability within the acceptable range defined by the R-1 standard. Similar findings have been reported in earlier studies on borate-treated composites, where low to moderate borate concentrations did not significantly affect linear expansion [42].

#### 3.1.6. Comparative Analysis of Mechanical and Physical Performance with Literature Data

The comparative analysis of percent variations in the mechanical and physical properties of treated particleboards (Table 2) reveals that the combined use of SBi and SBo results in moderate reductions in mechanical performance but significant improvements in dimensional stability. Specifically, the MOR and MOE decreased by 4–16%, while the IB exhibited slight reductions (−6 to −11%) compared to the control, indicating that chemical impregnation had a slight effect on the structural integrity of the boards. In contrast, the thickness swelling (TS) decreased by up to 11%, suggesting improved resistance to water absorption and dimensional changes. When compared to APP-, DAP-, or MAP-treated OSBs, which exhibited larger MOR and MOE reductions (−9% to −39%) and moderate improvements in TS (−5% to −22%) [34,43], the present SBi/SBo formulations produced less mechanical degradation, reflecting a milder effect on the wood matrix. Moreover, compared with boric acid–bark particle composites (P16-20 and P16-30) that displayed notable losses in stiffness and strength (−20% to −33%) but marked improvements in water resistance (up to −85%) [4], the current treatment achieved a better balance between mechanical retention and dimensional stability. Overall, in terms of mechanical and physical performance, the P150-50 formulation exhibited the most balanced performance, combining acceptable mechanical strength retention with enhanced dimensional stability, suggesting it as the most promising and sustainable alternative among the tested fire-retardant treatments.

### 3.2. Flammability and Thermal Properties

#### 3.2.1. Thermogravimetric Analysis (TGA)

**Figure 6:**
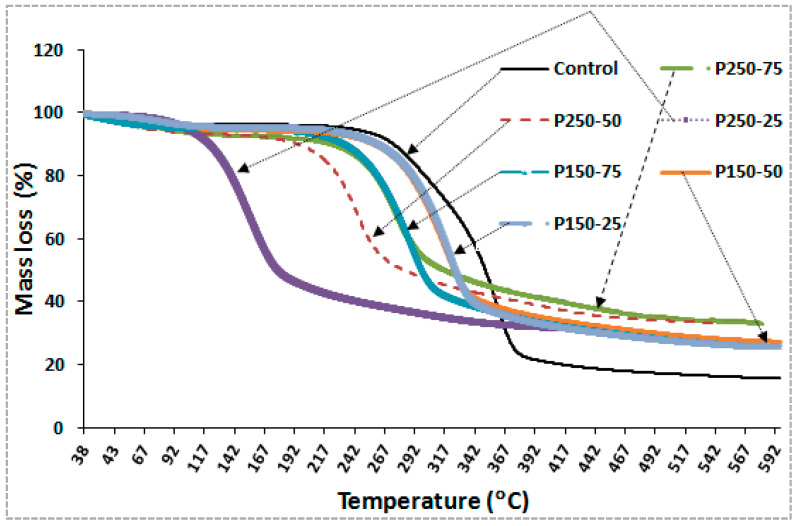
Thermogravimetric analysis (TGA) traces [P_c_ = control board; P_x−y_ = description of treating solution: X g of sodium bicarbonate and Y g of sodium borate].

The thermogravimetric analysis (TGA) curves (Figure 6) revealed consistent thermal degradation profiles for all boards, with clear differences between treated and untreated samples. The untreated control exhibited the highest total mass loss (83.1%) at 500 °C, leaving only 16.9% residual char, indicative of poor thermal stability and rapid devolatilization due to the absence of fire-retardant additives. In contrast, the P250-50 formulation (250 g SBi + 50 g SBo) exhibited the best thermal resistance, with a mass loss of 68.5% and a corresponding char yield of 31.5%, representing nearly a two-fold improvement over the control. This substantial increase in residue demonstrates the formation of a dense, thermally stable char layer that restricts oxygen diffusion and inhibits the release of flammable volatiles during decomposition.

Intermediate formulations, such as P250-25 and P150-50, also showed enhanced stability, with residual masses of 29–30%, confirming the beneficial role of both SBo and SBi. The improvement is attributed to the synergistic interaction between the two compounds: SBi decomposes to produce CO_2_ and Na_2_CO_3_, which dilutes combustible gases and contributes to early char nucleation, while SBo facilitates crosslinked borate–cellulose structures, reinforcing the char matrix and slowing down pyrolysis. These mechanisms collectively enhance thermal stability and align with previously reported effects of mineral-based fire retardants in lignocellulosic composites [27,32,44]. The superior performance of the P250-50 formulation underscores its optimal balance between chemical loading and matrix compatibility, making it a promising candidate for industrial-scale fire-resistant OSB panels.

#### 3.2.2. Mass Loss (ML): Tunnel and TGA Methods

**Figure 7:**
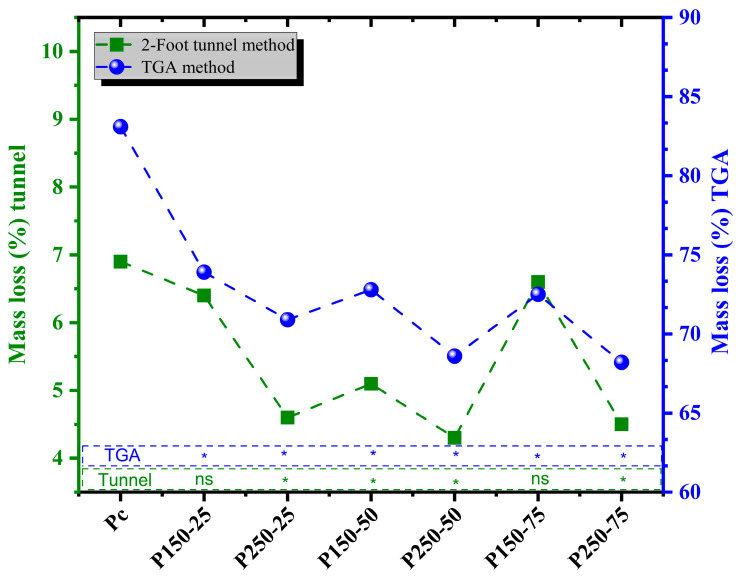
Mass loss (ML) tunnel and TGA methods [P_c_ = control board; P_x−y_ = description of treating solution: X g of sodium bicarbonate and Y g of sodium borate. The ML of each board type is compared to controls using Dunnett’s test. Results are presented in the histogram as either ns (non-significant) or * (significant)].

Results from both tunnel and TGA-based mass loss tests followed a consistent trend, reinforcing the reliability of the measurements (Figure 7). The control boards recorded the highest mass loss (6.9%) in the tunnel test, while the P250-50 treatment exhibited the lowest mass loss (4.3%), corresponding to a 38% reduction relative to the control. TGA results mirrored this trend, with total thermal mass loss decreasing from 83.1% (control) to 68.6% (P250-50).

ANOVA revealed no significant interaction between SBi and SBo for ML values, indicating that each compound independently contributes to the improvement in fire resistance. However, Dunnett’s test confirmed significant reductions in ML for all boards containing the highest concentrations of both additives (*p* < 0.05). These results demonstrate that the combined system effectively enhances thermal stability and fire retardancy, primarily through condensed-phase mechanisms involving char promotion and CO_2_ release, which act to suppress further mass loss.

Such behavior is consistent with prior studies, which have shown that inorganic flame retardants enhance the thermal resistance of lignocellulosic materials by forming protective barrier layers and modifying pyrolysis pathways [31,34,45]. In the present work, the strong agreement between tunnel and TGA measurements confirms that the dual SBi–SBo system exhibits robust and reproducible fire-retardant behavior under both thermal and flame-exposure conditions.

#### 3.2.3. Flame Spread Speed (FSS)

**Figure 8:**
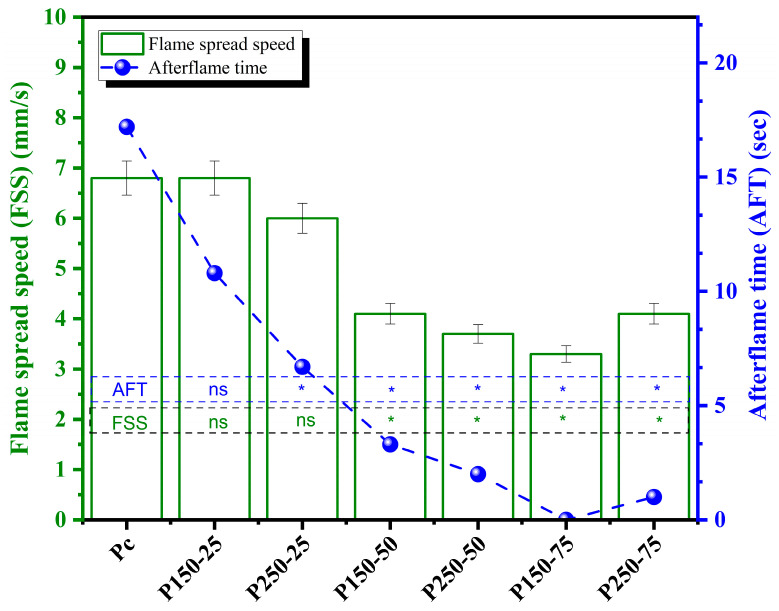
Flame spread speed (FSS) (mean values and standard deviation) and afterflame time (AFT) [P_c_ = control board; P_x−y_ = description of treating solution: X g of sodium bicarbonate and Y g of sodium borate. The FSS and AFT of each board type are compared to control using Dunnett’s test. Results are presented in the histogram as either ns (non-significant) or * (significant)].

The results indicate a clear retardation of flame propagation in particleboards treated with SBo and SBi. As shown in Figure 8, the flame spread speed (FSS) decreased progressively with increasing concentrations of the treatment salts, with boards treated at the highest concentrations (250 g SBi and 50–75 g SBo) exhibiting the lowest FSS values of 3.3–4.1 mm/s compared to 6.8 mm/s for the untreated control (Pc). Similarly, the after-flame time (AFT) was significantly reduced from 17.2 s in the control to as low as 0–3.3 s for the most heavily treated boards. These trends suggest that the incorporation of SBo and SBi into the particleboard matrix enhances its flame-retardant behavior, likely through a combination of char formation, endothermic decomposition, and release of non-combustible gases that inhibit flame propagation. Statistical analysis using Dunnett’s test confirmed that particleboards treated with the highest salt concentrations had significantly lower FSS and AFT values compared to the control, highlighting the effectiveness of these additives in improving fire performance. The observed reduction in both FSS and AFT demonstrates a dual benefit of the treatment, as it both slows the initial flame spread and limits continued combustion after ignition.

#### 3.2.4. Mechanistic Insights into the Flame Retardancy of SBo and SBi in OSB

The fire-retardant actions of SBi and SBo operate through two complementary mechanisms: condensed-phase and gas-phase processes, with additional contributions to dimensional stability [32,44,46].

In the condensed phase, SBi decomposes during heating into carbon dioxide (CO_2_) and sodium carbonate. The latter contributes to the formation of a compact, thermally stable char layer, which limits oxygen diffusion and acts as a physical barrier against heat transfer. Simultaneously, SBo transforms into borate salts with high thermal resistance, further reinforcing the integrity of this protective char. This synergistic char layer significantly delays ignition and reduces flame propagation.

In the gas phase, SBi releases CO_2_, which dilutes the local oxygen concentration and suppresses combustion intensity. In parallel, SBo releases boron-containing species capable of scavenging highly reactive radicals (H·, OH·), thereby interrupting the chain reactions responsible for flame propagation. This dual gas-phase action not only reduces flame spread speed but also shortens after-flame duration. Beyond fire performance, both additives contribute to dimensional stability. The presence of sodium salts within the wood matrix promotes partial pore filling and reduces capillary water uptake. This effect limits thickness swelling and enhances structural integrity under humid conditions.

Overall, the combination of condensed-phase char promotion, gas-phase radical quenching, and salt-induced dimensional stability contributes to the enhanced fire resistance and mechanical durability of the treated oriented strand boards. These mechanisms explain the substantial reduction in mass loss at 500 °C, the slower flame spread, and the preservation of board cohesion under fire exposure, particularly in the P250-75 and P250-50 formulations.

### 3.3. Statistical Analysis

The variance analysis (ANOVA) (Table 3) confirmed that both the main factors—SBi (MBi) and SBo (MBo)—as well as their interactions, significantly influenced several key board properties. MBi had a highly significant effect on internal bond strength (F = 33.55, *p* < 0.01), thickness swelling (F = 69.88, *p* < 0.01), and linear expansion (F = 18.30, *p* < 0.01), demonstrating the strong role of SBi in improving bonding and dimensional stability. Conversely, MBo showed significant effects on MOR (F = 6.82, *p* < 0.05), MOE (F = 7.09, *p* < 0.05), and especially on IB (F = 64.60, *p* < 0.01) and TS (F = 36.45, *p* < 0.01), highlighting its dual role in reducing strength while promoting fire resistance. The interaction term (MBi × MBo) was also significant for MOR (F = 5.06, *p* < 0.05), IB (F = 11.04, *p* < 0.01), and TS (F = 10.95, *p* < 0.01), confirming the synergistic but sometimes antagonistic interplay between the two salts. Orthogonal contrasts further clarified these trends: the linear effect of MBo (MBo-L) was extremely strong for IB (F = 114.36, *p* < 0.01) and TS (F = 68.15, *p* < 0.01), while quadratic effects (MBo-Q) were evident for IB and MOR, suggesting that performance responses were non-linear with respect to borate concentration. Blocking was effective in controlling nuisance variability, as only linear expansion showed a significant block effect (F = 4.31, *p* < 0.05).

Taken together, the statistical results demonstrate that: (i) SBi primarily drives bond integrity and dimensional stability, (ii) SBo strongly governs strength trade-offs and fire-retardant properties, and (iii) their combined application requires optimization to balance these contrasting effects. These findings justify the selection of P250-50 as the optimal formulation, as it aligns with the statistical evidence of positive MBi effects while minimizing the negative linear and quadratic impacts of MBo.

## 4. Conclusions

This study demonstrates that the dual impregnation of oriented strand board (OSB) strands with SBi and SBo represents a novel, halogen-free, and industrially compatible approach to flame retardancy. Unlike conventional treatments based on halogenated or phosphorus-containing additives, which often raise environmental and health concerns, this system relies on non-toxic, low-cost inorganic salts that can be easily integrated into existing OSB production lines without requiring process modification. The experimental results confirm the synergistic effect between SBi and SBo. Compared with the untreated control, the optimized formulation achieved a 38% reduction in mass loss, a greater than 50% decrease in flame-spread rate, and a twofold increase in residual char yield, indicating enhanced thermal stability and fire resistance. Moreover, mechanical testing revealed a 16% improvement in internal bond strength, while maintaining the moduli of elasticity and rupture within acceptable industrial ranges. Thus, the treatment does not compromise structural integrity. These findings validate the effectiveness of the SBi–SBo dual system as a sustainable and scalable alternative to traditional flame-retardant technologies. Furthermore, treatment not only enhances fire performance but also supports the transition toward environmentally responsible and halogen-free wood composites.

## Figures and Tables

**Table 1:** Composition of impregnation solutions used for strand treatment.

Code Sample	Solution Content of Impregnation		
	SBi (g)	SBo (g)	Water (L)
Pc ^1^	0	0	0
P150-25	150	25	4
P150-50	150	50	4
P150-75	150	75	4
P250-25	250	25	4
P250-50	250	50	4
P250-75	250	75	4

^1^ Control board (no impregnation).

**Table 2:** Percent variation in mechanical and physical properties of treated particleboards relative to reference samples.

Specimens	Fire Retardant Additive	MOR Change (%)	MOE Change (%)	IB Change (%)	TS Change (%)	Reference
OSB-APP	Ammonium polyphosphate (APP)	−39.5	−12.2	−27.4	−22.6	[34]
OSB-TBC	1,3,5-tris(2,3-dibromopropyl)-1,3,5-triazinane-2,4,6-trione (TBC)	−29.1	+14.5	+0.9	−73.9	[34]
OSB-(APP + TBC)	(APP + TBC)	−36.0	−7.3	−20.5	−29.2	[34]
OSB-(BX/BA)	Borax + boric acid- (BX/BA)	−5.6	−4.4	−4.9	−24.4	[43]
OSB-DAP	Diammonium phosphate (DAP)	−9.0	−13.2	−11.3	−5.3	[43]
OSB-MAP	Monoammonium phosphate- (MAP)	−13.5	−18.0	−7.4	−8.4	[43]
OSB-(BA/BP) P16-20	16% boric acid + 20% bark particles	−20.4	−19.7	34.6	−61	[4]
OSB-(BA/BP) P16-30	16% boric acid + 30% bark particles	−32.9	−27.1	30.9	−85.4	[4]
OSB-(SBi/SBo) P150-50	150 g sodium bicarbonate + 50 g sodium borate	−4.2	−3.6	−11.4	1.6	This work
OSB-(SBi/SBo) P250-50	250 g sodium bicarbonate + 50 g sodium borate	−15.8	−16.6	−6.82	−11.2	This work

**Table 3:** Summary of variance analysis (ANOVA) [DF = degree of freedom, MOE = modulus of elasticity, MOR = modulus of rupture, IB = internal bond, TS = thickness swelling, LE = linear expansion, FSS = flame spread speed, ML_tu_ = Mass loss (Tunnel method), WL_tga_ = weight loss (TGA method)].

Source of Variation		Physical and Mechanical Properties (F Values)					Fire-Retardant Performance (F Values)		
	Df	MOE	MOR	IB	TS	LE	FSS	ML_tu_	ML_TGA_
Block	2	3.85 ns	1.18 ns	0.16 ns	0.30 ns	4.31 *	0.89 ns	0.54 ns	0.23 ns
M_Bi_	1	0.08 ns	1.91 ns	33.55 **	69.88 **	18.30 **	0.03 ns	29.09 **	19.27 **
M_Bo_	2	7.09 *	6.82 *	64.60 **	36.45 **	1.98 ns	10.53 **	3.95 ns	2.20 ns
M_Bi_ × M_Bo_	2	4.06 ns	5.06 *	11.04 **	10.95 **	2.55 ns	0.89 ns	2.04 ns	0.24 ns
Contrasts									
M_Bi_	1	0.08 ns	1.91 ns	33.55 **	69.88 **	18.30 **	0.03 ns	29.09 **	19.27 **
M_Bo-L_	1	7.47 *	6.13 *	114.36 **	68.15 **	3.96 ns	16.87 **	0.04 ns	3.83 ns
M_Bo-Q_	1	6.70 *	7.50 *	14.83 **	4.76 ns	0.00 ns	4.20 ns	7.86 *	0.56 ns
M_Bi_ × M_Bo-L_	1	0.83 ns	2.22 ns	10.77 **	1.73 ns	3.17 ns	1.61 ns	0.24 ns	0.38 ns
M_Bi_ × M_Bo-Q_	1	7.30 *	7.91 *	11.30 **	20.16 **	1.94 ns	0.17 ns	3.85 ns	0.09 ns

Description of abbreviations: M_Bi_ = Mass of sodium bicarbonate in 4 L of water; M_Bo_ = Mass of sodium borate in 4 L of water; M_Bi_ × M_Bo_ = interaction between (M_Bi_) and (M_Bo_); M_Bo-L_ = linear effect of M_Bo_; M_Bo-Q_ = quadratic effect of M_Bo_; M_Bi_ × M_Bo-L_ = M_Bi_ versus linear effect of M_Bo_; M_Bi_ × M_Bo-Q_ = M_Bi_ versus quadratic effect of M_Bo_; ns = non-significant, * = significant at 0.05 probability level; ** = significant at 0.01 probability level.

## Data Availability

The original contributions presented in the study are included in the article, further inquiries can be directed to the corresponding author.
